# Dermatoses in international travellers seen at Bordeaux teaching hospital travel clinic, 2015–2018: a GeoSentinel‐based study

**DOI:** 10.1111/ced.14170

**Published:** 2020-02-17

**Authors:** R. Blaizot, E. Ouattara, M. C. Receveur, M. Mechain, T. Pistone, D. Malvy, A. Duvignaud

**Affiliations:** ^1^ Division of Tropical Medicine and Clinical International Health Department of Infectious Diseases and Tropical Medicine CHU Bordeaux Bordeaux France; ^2^ Univ. Bordeaux Inserm 1219 – Infectious Diseases in Lower Income Countries ISPED Bordeaux France

## Abstract

Skin disorders are frequent in travellers, but data vary between different studies. The objectives of the current study were to describe imported dermatoses in the Bordeaux GeoSentinel prospective database between August 2015 and March 2018. During the study period, 1025 travellers were seen in the clinic, 201 of them with dermatoses. Patients with skin disorders were more likely to be aged > 60 years (OR = 1.88, 95% CI 1.22–2.89), to be tourists (OR 3.04, 95% CI 2.03–4.55) and to have travelled to South America (OR = 2.18, 95% CI 1.29–3.67), and less likely to have sought pretravel advice (OR = 0.53, 95% CI 0.31–0.91). Skin bacterial infections (19.4%) and Zika virus infections (18.4%) were the most common dermatoses. Dengue fever and bacterial skin infections were the leading causes of hospitalization. The contribution of tropical diseases to imported dermatoses remains important. Lack of pretravel advice puts tourists at risk of significant diseases such as dengue fever, Zika virus and bacterial infections.

Skin disorders are one of the leading causes of illness in international travellers.^1^, ^2^, ^3^, ^4^, ^5^ However, information on these conditions is scarce. Additionally, the influence of the Zika outbreak on the spectrum of imported dermatoses in referral centres has not been studied. Our Bordeaux site joined the GeoSentinel Global Surveillance Network in 2013, providing prospective data on travel‐related illnesses for the entire Bordeaux metropolitan area (about 1 195 000 inhabitants). The aim of this study was to update data concerning dermatoses in French travellers through the GeoSentinel system.

## Report

All patient records from August 2015 to March 2018 were extracted. Patients with dermatoses were defined as those consulting for primary skin disorders or systemic diseases with significant dermatological symptoms. All other patients were classified as nondermatological. Potential associations between patient and travel characteristics and the occurrence of dermatoses were explored using logistic regression models.

During the study period, 1025 patients were recorded, of whom 201 (19.6%) were referred for a skin disorder. In multivariate analysis, compared with patients with nondermatological diseases, patients with skin disorders were more likely to be aged > 60 years (OR = 1.88, 95% CI 1.22–2.89, *P* < 0.001), to be tourists (OR = 3.04, 95% CI 2.03–4.55, *P* < 0.001) and to have travelled to South America (OR = 2.18, 95% CI 1.29–3.67, *P* < 0.001) and less likely to have sought pretravel advice (OR = 0.53, 95% CI 0.31–0.91, *P* < 0.01).

**Figure 1:**
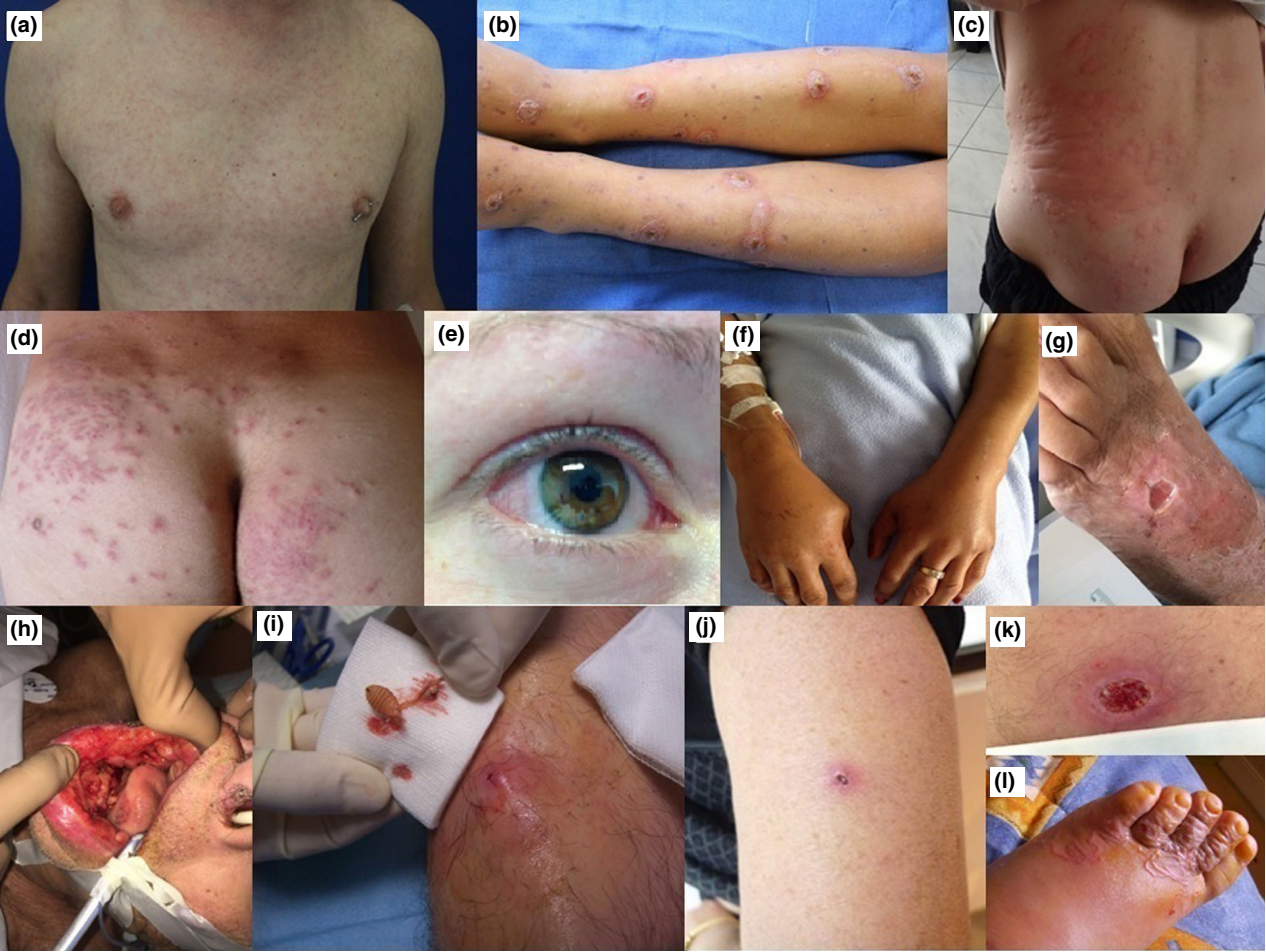
(a–l) Clinical presentations of several imported dermatoses in a cohort of returning travellers, Bordeaux, 2015–2018, top to bottom, left to right: (a) papular rash after Zika virus infection acquired in Nicaragua; (b) ecthyma of the lower limbs after sleeping on a Colombian beach; (c) urticarial rash during a *Strongyloides stercoralis* infestation; (d) hookworm‐related folliculitis; (e) conjunctivitis during Zika virus infection; (f) Calabar oedema of both hands after infestation with the filarial nematode *Loa loa*; (g) clean, geometric ulcer of cutaneous diphtheria in a woman living in a derelict beach hut in Senegal; (h) mouth ulcerations caused by *Histoplasma capsulatum*; (i) furuncle‐like lesion caused by a larva of *Dermatobia hominis*; (j) black eschar during a rickettsial infection; (k) undermined borders in a Buruli ulcer; and (l) hookworm‐related cutaneous larva migrans with serpiginous track.

Of the patients with skin disorders, 55.7% were women and 44.3% were men, mean age was 41.2 years and mean trip duration was 20 days. The most frequent purposes of travel were tourism (69.2%), migration (8.5%), business (7.9%) and visiting friends/relatives. The most frequent regions of exposure were South America/Caribbean (39.7%), sub‐Saharan Africa (27.9%) and Asia (23.4%). Inpatient treatment was required for 13.4% of patients, mostly for bacterial skin infections (44.4% of hospitalizations) and dengue (22.2%). Zika virus infections represented a large percentage (18.4%) of the recorded disorders, and were observed in patients returning from the French West Indies (75.7% of cases), Latin America (10.8%) and the non‐French Caribbean islands (13.5%). Two patients sexually transmitted Zika virus to their respective partners after returning to France. Some diseases were less commonly reported in travellers, such as Buruli ulcer, American histoplasmosis, ciguatera, leptospirosis, gnathostomiasis, toxocariasis and rickettsial infections (Table 1). Bacterial infections were the leading causes of skin disorders in travellers returning from Asia and Africa (0.26 and 0.23 cases per traveller respectively). Filariasis and leprosy were mostly observed in migrants from Africa (0.05 cases/traveller), while Zika represented the most frequent disorder in South America (0.49 cases/traveller). Dengue fever was the second most frequent disease in travellers returning from Asia (0.23 cases/traveller). The clinical pictures of some of these diseases are presented in Fig. 1.

**Table 1:** Description of dermatological disorders and pathogens in the study population in a Bordeaux teaching hospital travel clinic during the period 2015–2018.

Type	*n* (%)
All skin disorders	201 (100)
Infection‐related skin disorders	159 (79)
Others	42 (21)
Bacterial	50 (25)
Cellulitis/ecthyma/erysipela	39 (78)
*Streptococcus pyogenes*	2
*Salmonella enteritidis*	1
*Staphylococcus aureus*	9
*Corynebacterium diphtheriae*	1
Unknown	26
Mycobacteria	5 (10)
*Mycobacterium ulcerans*	1
*Mycobacterium tuberculosis*	1
*Mycobacterium leprae*	3
*Rickettsia*	4 (8)
Leptospirosis	2 (4)
Parasitic	36 (18)
Creeping eruption	19 (53)
Hookworm‐related	15
Gnathostoma	1
Unknown	3
Myiasis	5 (14)
*Dermatobia hominis*	2
*Cordylobia anthropophaga*	3
Scabies	5 (14)
Filaria	4 (11)
*Loa loa*	1
Unknown	3
*Toxocara*	2 (6)
Schistosomiasis	1 (3)
Fungal	12 (6)
Dermatophytosis	10 (84)
*Histoplasma capsulatum*	1 (8)
Mycetoma	1 (8)
Other	42 (21%)
Arthropods	18 (43%)
Flea	1
Tsetse fly	1
Tick	4
Unknown	12
Other animals	5 (12%)
Monkey	1
Other terrestrial	1
Jellyfish	1
Scorpion fish	1
Unknown marine	1
Noninfectious/not animal‐related	19 (45)

Dermatological disorders made up almost 20% of all diseases in this population, a proportion close to that of previous studies.^1^, ^2^, ^3^ Most cases were treated as outpatients (86.6%). However, dengue fever and bacterial skin infections frequently required inpatient treatment. As previously reported,^3^ seeking pretravel advice was statistically less likely for patients with skin disorder, suggesting a likely method for prevention. Similarly, our data support previous results of the association between skin disorders and tourism as a purpose of travel.^3^ However, we found an unusual association with age > 60 years, but we did not find a significant association with female sex, which has been reported in other cohorts.^1^, ^2^

A decline in the proportion of strictly tropical diseases among imported dermatoses has been suggested.^2^, ^3^ However, tropical diseases accounted for half of all conditions in our study. Beside possible variations in behaviours, the Zika outbreak offers an explanation. We also identified several cases of unusual imported diseases, particularly in migrants. Arboviruses were responsible for a large percentage (27.8%) of the recorded conditions. Dengue fever with rash was one of the major risks in Asia, owing to the occurrence of the rainy season during the peak travel period (July–November).^6^ This study offers a clear view of how the clinical epidemiology of dermatoses in travellers can be affected by emerging diseases such as Zika virus. A follow‐up of several months is necessary for young couples, owing to the potential persistence of Zika virus in semen,^7^ and the fact that two of our patients infected their partners. Considering a significant rate of subclinical infections,^8^ these findings support large‐scale screening of poorly symptomatic travellers.

This study highlights the clinical burden and wide spectrum of clinical presentations caused by imported dermatoses. Travellers should be encouraged to seek pretravel advice, with special care given to the prevention of dengue and bacterial infections, owing to their potential severity. Dermatologists seeing travellers should be familiar with the symptoms associated with tropical diseases such as *Mycobacterium ulcerans* infection, filariasis, toxocariasis or leprosy.

**Learning points**

- Although most skin disorders are managed in outpatient consultation, dengue fever and bacterial skin infections are significant causes of hospitalization.
- Travellers to Asia were more at risk of dengue fever, while Zika represented the main risk in America during the study period.
- Tourists, those travelling to South America and elderly travellers were found to be more at risk of dermatological disorders than other travel‐related illnesses.
- Patients with imported dermatoses were less likely than patients with other imported illnesses to seek pretravel health advice, and improved prevention would be beneficial to manage these dermatological disorders.
- The Zika virus outbreak had a significant impact on a cohort of patients with imported dermatoses and highlights the importance of emerging tropical diseases for dermatologists seeing travellers returning from endemic or epidemic areas.
- Dermatologists seeing travellers should be aware of the risk of filariasis and leprosy.
